# Scaling Up Forest Vision with Synthetic Data

**DOI:** 10.1007/s11263-026-02923-y

**Published:** 2026-07-11

**Authors:** Yihang She, Andrew Blake, David Coomes, Srinivasan Keshav

**Affiliations:** 1 Department of Computer Science and Technology, University of Cambridge, 15 JJ Thomson Avenue, Cambridge, CB3 0FD United Kingdom; 2 Clare Hall, University of Cambridge, Herschel Road, Cambridge, CB3 9AL United Kingdom; 3 Department of Plant Sciences, University of Cambridge, Downing Street, Cambridge, CB2 3EA United Kingdom

**Keywords:** 3D Vision, Synthetic Data, Tree Segmentation, LiDAR Simulation, AI for Science

## Abstract

Accurate tree segmentation is a key step in extracting individual tree metrics from forest laser scans, and is essential to understanding ecosystem functions in carbon cycling and beyond. Over the past decade, tree segmentation algorithms have advanced rapidly due to developments in AI. However, existing public 3D forest datasets are not large enough to build robust tree segmentation systems. Motivated by the success of synthetic data in other domains such as self-driving, we investigate whether similar approaches can help with tree segmentation. In place of expensive field data collection and annotation, we use synthetic data during pretraining, and then require only minimal, real forest plot annotation for fine-tuning. We have developed Cambridge Arboreal Modelling Panoptic 3D (CAMP3D), a new synthetic data generation pipeline to do this for forest vision tasks, integrating advances in game engines with physics-based LiDAR simulation. Using CAMP3D, we have produced a comprehensive, diverse, annotated 3D forest dataset on an unprecedented scale. Extensive experiments with a state-of-the-art tree segmentation algorithm and a popular real dataset show that our synthetic data can substantially reduce the need for labelled real data. After fine-tuning on just a single, real, forest plot of less than 0.1 hectare, the pretrained model achieves segmentations that are competitive with a model trained on the full scale real data. We have also identified critical factors for successful use of synthetic data: physics, diversity, and scale, paving the way for more robust 3D forest vision systems in the future. Our CAMP3D pipeline and the resulting dataset are available at https://github.com/yihshe/CAMP3D.git.

## Introduction

Understanding forest dynamics and monitoring their status have long been a priority in the Sustainable Development Goals of the United Nations (Swamy et al., 2018). Forests can be monitored at different spatial scales from ground surveys to space missions, with varying trade-offs between accuracy, cost, and spatial coverage (Dubayah et al., 2020; Liang et al., 2022; Lines et al., 2022). Forest monitoring at a close range (Liang et al., 2022) has become increasingly important for precise forest management. It also offers ground reference data to calibrate satellite remote sensing (Lang et al., 2022).

**Fig. 1:**
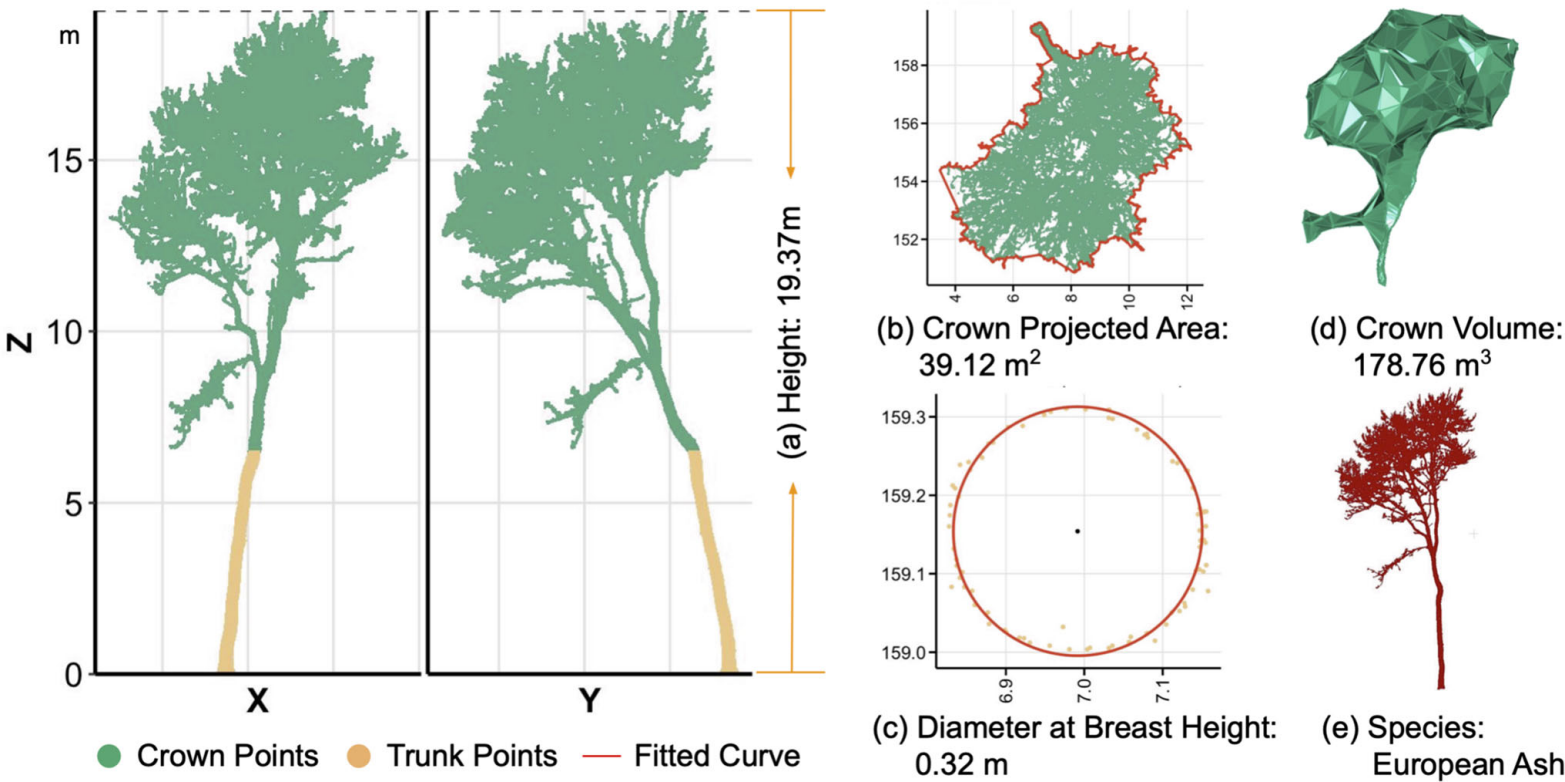
Individual tree segmentation is essential for deriving key forest properties in ecology. This figure shows examples of metrics derived from individual tree segmentation: canopy height and diameter at breast height for biomass and carbon estimation, and crown area, crown volume, and species composition for understanding forest structure, biodiversity, and ecosystem functions

For close-range forest sensing to obtain metrics for individual trees, the typical workflow involves obtaining point clouds of forests through laser scanning and segmenting individual trees. The next step is to extract the properties of interest, such as above-ground biomass (Coomes et al., 2017), species (Puliti et al., 2024), tree health (Chan et al., 2021), crown volume, canopy height and diameter at breast height (DBH) (Liang et al., 2019), among others. Accurate measurement of these properties is essential for understanding key ecosystem functions related to carbon cycling and beyond (Fig. 1). For example, canopy height and DBH are fundamental for quantifying forest metabolic scaling and allometry, which underpin the estimation of above-ground biomass (Chave et al., 2009). The shape of the canopy surface and the volume of the crown reveal competitive light dynamics, providing indicators of tree health and reflecting the role of the forest in the energy cascade of ecosystems (Purves et al., 2007). Metrics derived from canopy surfaces are also critical for studying canopy–atmosphere gas exchange, including the uptake and release of greenhouse gases such as methane and carbon dioxide (Gauci et al., 2024). The accurate segmentation of individual trees is a key bottleneck in the entire pipeline.

The choice of algorithm plays a crucial role in building a robust tree segmentation system. Over the past decade, tree segmentation has changed from the use of the classical region-growing algorithm (Coomes et al., 2017) to increasingly complicated neural network architectures (Ball et al., 2022; Straker et al., 2023; Wielgosz et al., 2023, 2024; Xiang et al., 2024). It also expanded from 2D image operations (Ball et al., 2022; Coomes et al., 2017; Straker et al., 2023) to the processing of 3D point clouds (Wielgosz et al., 2023, 2024; Xiang et al., 2024). All of this progress has refined the segmentation of trees for a more accurate retrieval of forest properties as discussed above.

The increasing complexity of tree segmentation algorithms also requires more data and computation for a stronger and more generalisable model performance (Sutton, 2019). Despite extensive data collection efforts (Calders et al., 2022; Puliti et al., 2023; Weiser et al., 2022), the available 3D forest dataset for the development of tree segmentation algorithms still falls short of the scale typically required for robust machine learning (Uddin & Lu, 2024). This is not surprising given that forest scanning requires field work that is limited by multiple factors, such as site accessibility, weather conditions, and seasons. In addition, the intricate canopy structures of forests make it challenging and time consuming to delineate the semantic labels (classifying each point by category, e.g., stem, branch, ground) and instance labels (assigning unique identifiers to individual trees). For example, Puliti et al. (2023) report that just annotating 2.79 hectares of forest plots takes two skilled researchers six months. Wielgosz et al. (2024) suggest that the scarcity of data has become a bottleneck for further improvement of algorithm performance.

Recent advances in graphics technology enable synthetic data generation to tackle data scarcity in other vision domains (Gaidon et al., 2016; Roberts et al., 2021; Shotton et al., 2011; Sklyarova et al., 2023). In self-driving vehicles, the use of synthetic data has become a key factor for the rapid development of this field (Song et al., 2023). In the context of forest monitoring, procedural foliage generation in graphics engines such as Unreal Engine (UE) has allowed the creation of forest scenes on a large scale (Holmberg, 2025). Researchers have repurposed game scenes to render synthetic 2D images for the development of tree segmentation algorithms (Feng et al., 2025; Grondin et al., 2022; Lu et al., 2024). Open source graphics engines such as Blender enable the creation of simulation packages tailored to natural scenes (Liu et al., 2021; Winiwarter et al., 2022). Simulators such as the Heidelberg LiDAR Operations Simulator (HELIOS) (Winiwarter et al., 2022) resemble the real-world laser scan process during a forest survey.

Synthetic data can significantly reduce the effort to collect data in the field and then to annotate the data, as many existing synthetic forest datasets have demonstrated (Bryson et al., 2024; Grondin et al., 2022; Lu et al., 2024; Wang, 2020). Nevertheless, its effectiveness depends on whether an algorithm trained on the synthetic data can generalise well to the real world. A key factor is the diversity of synthetic data on large scales: with enough diversity, synthetic data could allow the algorithm trained on it to generalize to real-world forests (Prakash et al., 2019; Tobin et al., 2017), and with large-scale data, neural networks can benefit from adequate computations for effective weight and bias learning (Sutton, 2019). Under this assumption, the learning paradigm for a forest vision algorithm would involve pretraining on synthetic data, followed by fine-tuning on real data from the region of interest. This approach, similar to meta-learning (Finn et al., 2017), is expected to enable the pretrained algorithm to generalize effectively to real-world scenarios, even when only a limited number of labelled samples are available.

Although several synthetic forest datasets have been developed using advanced simulation tools such as HELIOS (Winiwarter et al., 2022), most are of relatively small scale and limited in structural diversity. For example, Wang (2020) manually created a 0.16 ha plot (40 m × 40 m) containing  30 trees of a single species, while Bryson et al. (2024) generated a plot of  400 trees in a regularly spaced, low-variability layout. The recently introduced Boreal3D dataset (Liu et al., 2025) is the largest to date, with 1,000 plots (each 20 m × 20 m) derived from real-world TLS scans of six coniferous plots in Finland— totalling 40 ha but covering only three coniferous species.

In contrast, we introduce the Cambridge Arboreal Modelling Panoptic 3D (CAMP3D) pipeline and dataset. Our approach sources forest scenes from the gaming industry and regenerates them using procedural foliage generation, originally developed in Unreal Engine but, as we find, grounded in ecological forest-growth principles. This enables the creation of structurally diverse forest scenes by leveraging prebuilt forest scenes and tree assets, repurposed to simulate point clouds for forest vision tasks. We then integrate these scenes with the HELIOS simulator to produce physically-based point clouds with accurate semantic and instance labels. The resulting data set spans 12 forest scenes over 75 ha, covering both coniferous and deciduous types, and exceeds the scale and diversity of real dataset such as Wytham Woods (1.4 ha) (Calders et al., 2022) and FOR-Instance (2.79 ha) (Puliti et al., 2023) and synthetic dataset such as Boreal3D (40 ha, limited diversity) (Liu et al., 2025).

Using our CAMP3D dataset, we experiment with the learning paradigm of pretraining followed by fine-tuning. We pre-train a state-of-the-art 3D tree segmentation algorithm (Xiang et al., 2024) on our synthetic dataset and validate it on a widely used real-world data set for tree segmentation (Puliti et al., 2023). Experiments show that the algorithm pretrained with our synthetic data gets significant improvement than training from scratch on a few real plots. Fine-tuning the pretrained algorithm on just one forest plot achieves segmentation accuracy comparable to full-scale real data, although this plot can represent as little as 2.2% of the labelled dataset. In summary, our contributions include:

- a synthetic data generation pipeline for forest vision tasks that combine advances in game engines and physics-based simulation;
- a comprehensive, diverse, and large-scale synthetic dataset of 3D point-clouds for tree segmentation tasks;
- experiments showing the effectiveness of the CAMP3D dataset, and revealing that physics-based simulation, scene diversity, and large dataset scale are all crucial for synthetic data to be effective for training.

## Related Work

### Close-range sensing of forest

Close-range sensing in forest monitoring involves using sensors from a few to several hundred meters away to gather detailed, contact-free data on trees and vegetation (Liang et al., 2022). Compared to remote sensing, close-range sensing of forest offers more fine-grained monitoring of forest status (Lang et al., 2023; Liang et al., 2022). It covers a wide range of platforms and sensors from laser scanners to RGB cameras, resulting in different data representations (Okura, 2022). In terms of sensor, laser scans have become a dominating sensing method in this field, and point clouds are the most common data representations. These sensing methods can be attributed to different platforms, such as terrestrial laser scanning (TLS) (Lines et al., 2022), airborne laser scanning (ALS) (Coomes et al., 2017), and unmanned aerial vehicle laser scanning (UAV-LS) (Nesbit & Hugenholtz, 2019). Each platform presents varying levels of trade-offs between accuracy and cost. TLS offers fine-grained information about tree structures; however, it has low spatial coverage and is expensive to operate (Lines et al., 2022). ALS can cover relatively large areas, but has low resolution and accuracy (Coomes et al., 2017). Recently, UAV advancements have provided a low-cost, high-resolution alternative to terrestrial and airborne platforms, gaining popularity in forest monitoring (Ball et al., 2022; Flynn, 2024; Nesbit & Hugenholtz, 2019). Accurate segmentation of individual trees is the key step to obtaining the properties of interest at an individual tree level (Chan et al., 2021; Coomes et al., 2017; Liang et al., 2019; Puliti et al., 2024).

### Tree segmentation algorithms and datasets

Tree segmentation algorithms have made outstanding progress in recent years due to the fast development of AI (Straker et al., 2023; Wielgosz et al., 2023, 2024; Xiang et al., 2023, 2024). Compared to segmenting trees from 2D images (Ball et al., 2022; Straker et al., 2023), segmenting trees from 3D point clouds can offer more accurate results, especially in dense canopies (Xiang et al., 2024). Moreover, 3D segmentation enables a wider range of tasks such as estimating crown volume using point clouds of individual trees instead of just their detected tree crowns (Liang et al., 2018, 2019). In terms of 3D tree segmentation, the state-of-the-art is ForAINet, developed by Xiang et al. (2024). It performs sparse 3D convolution on point clouds and has multiple task heads designed for both instance and semantic segmentation. SegAnyTree (Wielgosz et al., 2023) employs the same architecture, but generalises the algorithm to different sensors by incorporating UAV and mobile laser scans, and downsampling dense point clouds to simulate airborne laser scans. The development and evaluation of algorithms are contingent on 3D forest point clouds with quality annotations. Only a few of such datasets are publicly available (Calders et al., 2022; Puliti et al., 2023; Weiser et al., 2022). For example, the Wytham Woods dataset (Calders et al., 2016) covers approximately 1 hectare of TLS scans of deciduous forests in southern England with instance segmentation labels. The FOR instance data set (Puliti et al., 2023) is a UAV-LS data set that covers five regions with instance and semantic segmentation labels, totalling 2.79 ha. In fact, both ForAINet (Xiang et al., 2024) and SegAnyTree (Wielgosz et al., 2024) are developed using the FOR-instance dataset (Puliti et al., 2023).

**Fig. 2:**
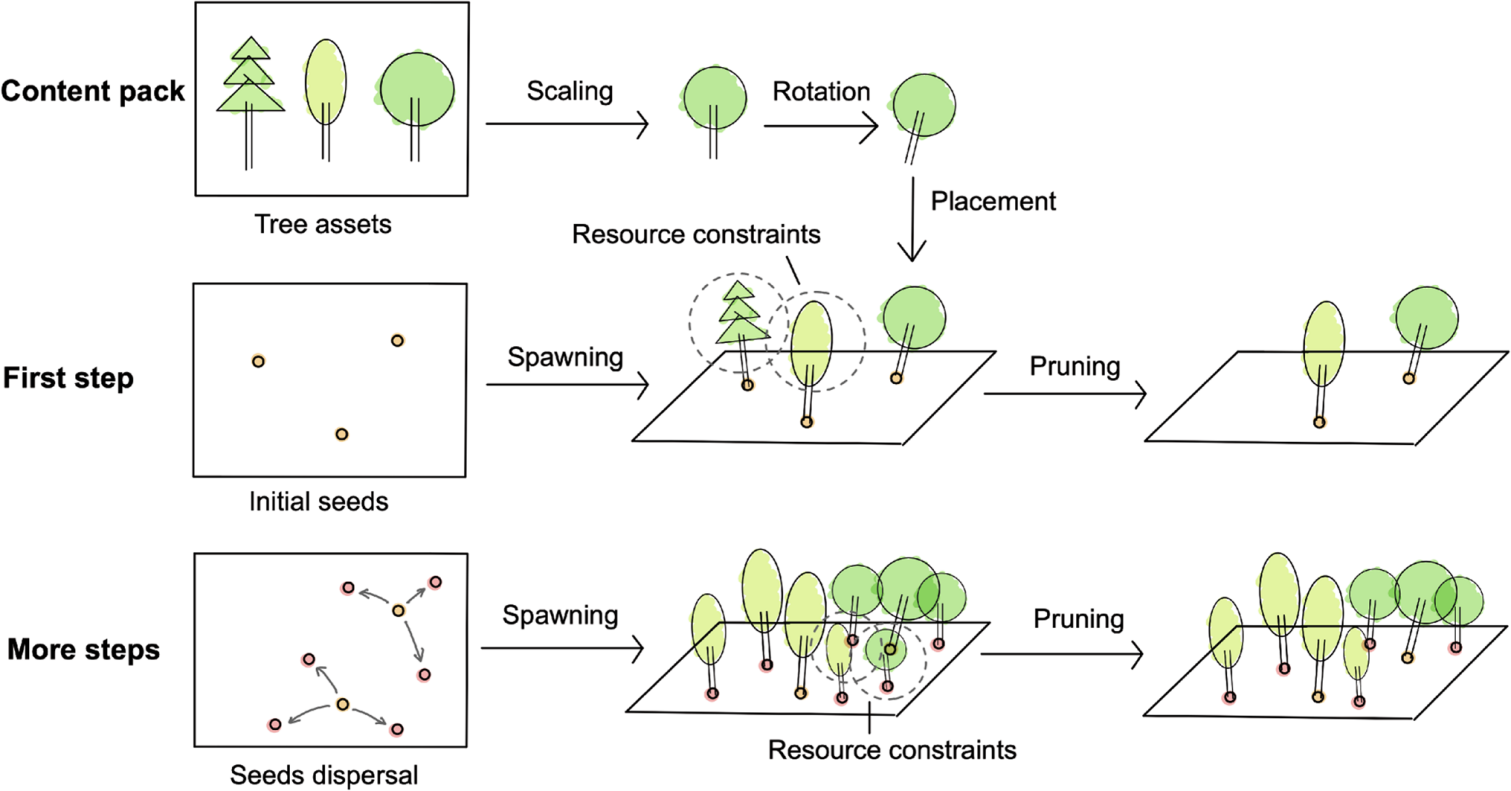
Photorealistic forest scenes can be generated, at large scale, with a limited set of tree models, using procedural foliage generation. All forest scenes we have collected from Unreal Engine were generated using this algorithm. Although developed and used mainly in the game industry, this algorithm has roots in ecological theory on forest growth (Pacala et al., 1996; Purves et al., 2007)

### Synthetic data and its applications

Synthetic data is extensively used in fields like human pose estimation (Shotton et al., 2011; Sklyarova et al., 2023), self-driving vehicles (Gaidon et al., 2016), and indoor scene understanding (Roberts et al., 2021) to combat real-world data scarcity. Its success is benefited from recent advances in graphics engines and simulators built on top of them (Bondi et al., 2018; Dosovitskiy et al., 2017; Liu et al., 2021; Winiwarter et al., 2022). In particular, Unreal Engine (UE) offers advanced rendering and procedural foliage generation for easy creation of large-scale forest scenes (Holmberg, 2025). Synthetic data sets have been created for 2D tree segmentation based on this feature (Feng et al., 2025; Grondin et al., 2022; Lu et al., 2024). Using UE and AirSim simulators Bondi et al. (2018), Feng et al. (2025) created the Synthetic Photorealistic Arboreal Dataset (SPREAD) with various forest scenes for training the 2D trunk segmentation algorithm. In parallel to UE-based simulation, some simulators have been built on Blender, which is open source and more customisable. Infinigen (Liu et al., 2021) focuses on rendering synthetic data for natural scenes in Blender, mainly for 2D scenarios. HELIOS (Winiwarter et al., 2022) is a simulator specifically designed for cross-platform forest laser scanning, meeting our requirements. This work introduces a novel data pipeline bridging UE’s advanced scene generation and HELIOS’s 3D LiDAR forest survey simulation. While we focus on generating synthetic data to improve machine learning algorithms, existing work has also used synthetic data for other studies of forests. These include comparing simulations of different sensor types with real-world laser scans (Esmorís et al., 2024; Schäfer et al., 2023) and simulating the forest radiative transfer process (Liu et al., 2022). In some of the studies available that generate synthetic laser scans for training tree segmentation algorithms (Bryson et al., 2024; Wang, 2020), forest stands are typically built manually. These are available on a small scale, such as a virtual 40 m × 40 m plot with 30 trees (Wang, 2020).

## Methods

### Synthetic data generation pipeline

We generate forest scenes for simulation using Unreal Engine’s procedural foliage generation algorithm ($\mathcal {A}_{\text {PFG}}$; see Fig. 2), which enables large-scale 3D forest generation from a limited set of foliage models. While the Unreal Engine community provides many pre-built scenes and asset packs, these are designed mainly for gaming applications and often contain non-forest elements or unsuitable layouts. We therefore regenerate the forest component within a predefined 250 m × 250 m plot, applying $\mathcal {A}_{\text {PFG}}$ with only tree foliage generators; if only tree assets are provided, we first define the foliage generators from these assets in order to run $\mathcal {A}_{\text {PFG}}$.

**Table 1:** Procedural Foliage Parameters for Coniferous Forests. Most of these parameters have physical meanings regarding how seeds of a tree can disperse and how the corresponding trees can be spawned at the overstory level (canopy trees) and the understory level (samplings or shrubs)

Parameter	Overstory	Understory
Initial Seed Density	3.0	2.0
Collision Radius	250–300 cm	50–100 cm
Shade Radius	400–500 cm	50 cm
Procedural Scale	0.8 – 1.2	0.5 – 1.0
Average Spread Distance	1500 cm	800 cm
Spread Variance	500 cm	300 cm
Num Steps	2	2
Max Age	3	2

This procedural foliage generation algorithm ($\mathcal {A}_{\text {PFG}}$), despite having originated from the game industry, is broadly consistent with ecological theory of forests originating and forming canopy structure through light and resource competition (Hallé & Oldeman, 1970; Pacala et al., 1996; Purves et al., 2007): given a bare plot of land, starting from seeds, trees grow and expand to fill all available space, but subject to constraints on light, represented by collision and shade radius in this algorithm. After enough years, or simulation steps, the scene reaches a state of equilibrium — a closed canopy — while still respecting individual space requirements. At a high level, the algorithm iteratively spawns and prunes foliage in simulation steps. Table 1 summarises some key parameters for procedural generation and examples of parameter ranges that can be used for coniferous forests. More details regarding these parameters can be found in Section A.2.

We built a diverse, simulation-ready dataset from selected and adapted forest scenes in readily available datasets and asset packs. First, five natural forest scenes come from the SPREAD dataset (Feng et al., 2025), originally developed for generating 2D images for trunk segmentation. The dataset contains six natural forests, four urban tree scenes, and one plantation. One natural forest scene (“Burned Wood”) is excluded, leaving three deciduous forests, one redwood forest, and one rainforest. In its original form, each SPREAD forest scene covers a 1000 m × 1000 m area constructed from only ten unique 100 m × 100 m tiles—a common default setting in game scene generation. While adequate for gaming applications, this design produces repeated spatial patterns, leading to redundancy in simulated data. To avoid this limitation, we regenerate each selected scene in a 250 m × 250 m (6.25 ha) plot with unique layouts containing only tree vegetation.

The SPREAD collection lacks European coniferous forests and has limited European deciduous coverage so we supplement it with seven additional forest scenes from the Unreal Engine Marketplace. The scenes are selected for their ecological relevance, specifically European coniferous and deciduous forests, and for the availability of high-quality tree assets at the time of collection. More scenes could be incorporated if suitable assets become available. In total, the dataset comprises twelve adapted forest scenes, covering 75 ha.

**Fig. 3:**
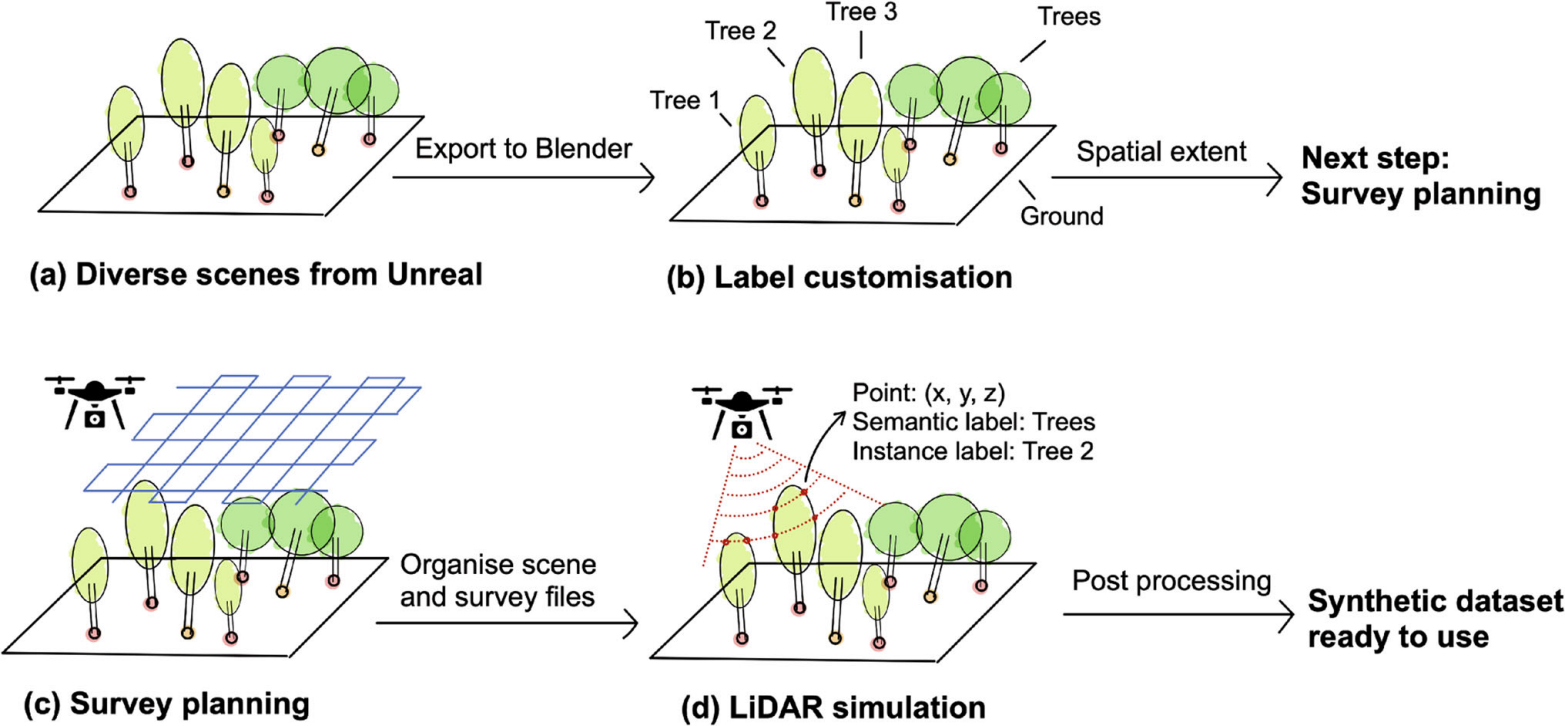
Synthetic data generation pipeline. A forest scene is processed: (**a**) adding files to Blender, (**b**) customizing instance and semantic labels, (**c**) planning a UAV flight path and setting up a virtual laser scanner, and (**d**) simulating a LiDAR survey

The HELIOS UAV simulator (Winiwarter et al., 2022) is then used in a pipeline (Fig. 3) to generate laser scans and annotated point clouds. For details of the configuration of simulation software see Section A.1.

**Table 2:** Synthetic data covers 12 forest scenes for a wide range of forest types, totalling 75 hectares. Forest scenes of the first five entries are adapted from the SPREAD dataset (Feng et al., 2025). The generated data is tiled into roughly 50 m × 50 m plots and split into train, test, and validation sets

Scene Name	Tree Species	Plots			points/m^2^
		Train	Val	Test	
Deciduous1	Birch	17	4	4	2092.687
Deciduous2	Beech, Oak	17	4	4	1414.716
Deciduous3	Ash, Linden	17	4	4	1638.955
Rainforest	Alii Fig, Palm, Fern, Rubber Fig, Umbrella	17	4	4	1593.875
Redwood	Sequoia	17	4	4	1120.249
Coniferous1	Scots Pine, Silver Birch	17	4	4	1693.415
Coniferous2	Scots Pine, Mountain Ash	17	4	4	1576.841
Coniferous3	Fir, Spruce	17	4	4	1944.650
Coniferous4	Scots Pine, Hornbeam	17	4	4	1679.339
Deciduous4	Beech, Hazel, Norway Maple, Ash	17	4	4	1604.083
Deciduous5	Black Alder, Hazel, Hornbeam, Ash	17	4	4	1716.359
Deciduous6	Hornbeam, Hazel, Linden	17	4	4	1767.841

**Fig. 4:**
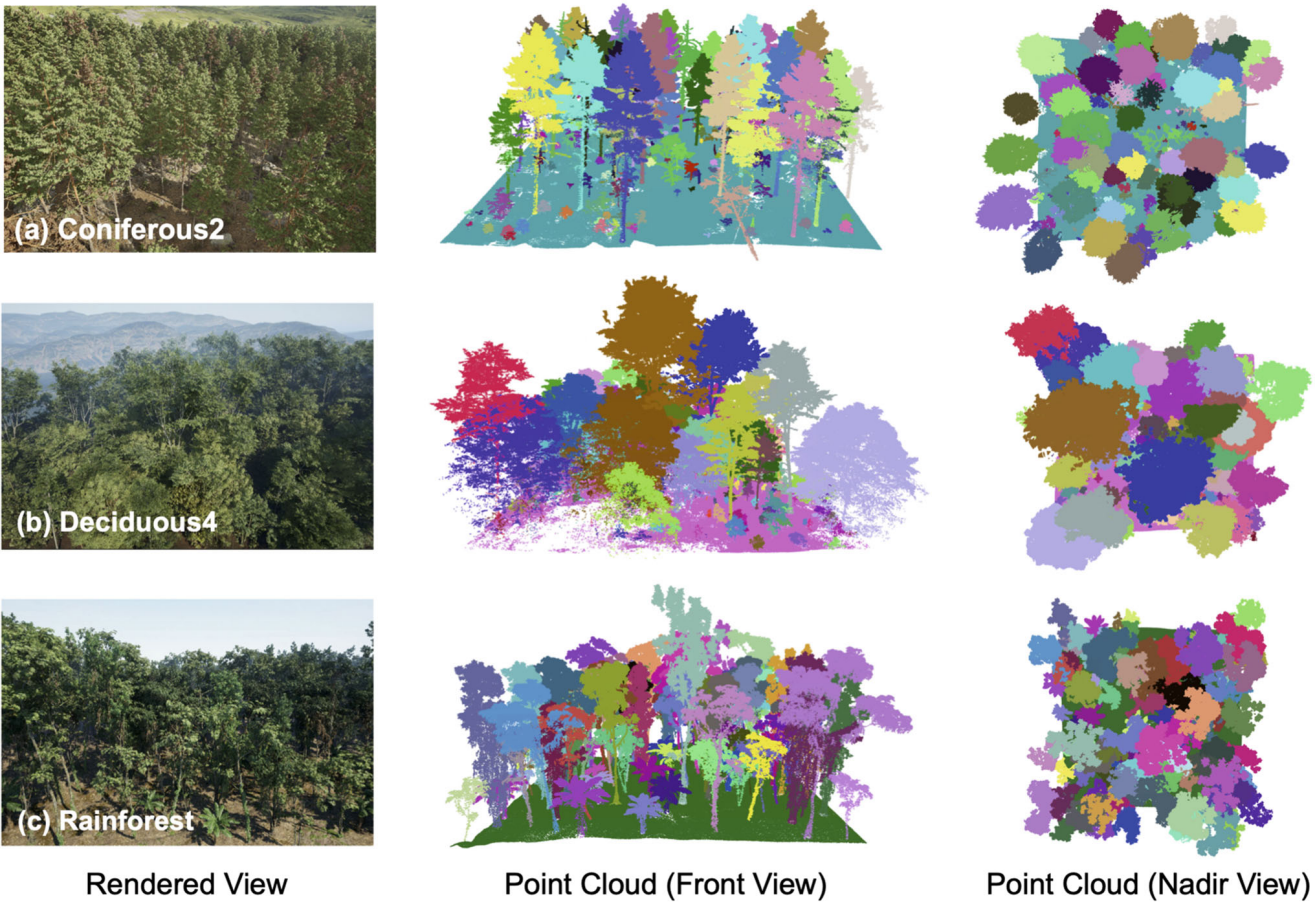
The synthetic data is diverse, physics-based, and available at large scale. Using Unreal Engine, large-scale, photorealistic forest scenes are created using gaming assets and procedural foliage. Then the pipeline simulates laser scans to produce synthetic point clouds with instance labels (shown here in colours). The 50m × 50m tiled plots are ready for machine learning

### Generated synthetic dataset

The synthetic data generation pipeline simulates UAV laser scans for 12 forest scenes — 5 adapted from the original SPREAD collection, and 7 new scenes (Table 2). The dataset includes 4 coniferous and 6 deciduous scenes, focusing on European forests (Fig. 4). Two additional scenes, Rainforest and Redwood, are retained from the original SPREAD collection for completeness, but are not our main regional focus. The data set is prepared for machine learning by merging point clouds from each scene, tiling them into 50m × 50m plots, and splitting them into training (70%), validation (15%) and test sets (15%). All plots have a point density greater than 1000 $\text {points/m}^2$, adequate for effective tree segmentation learning (Wielgosz et al., 2024).

The resulting synthetic data has not only class labels but also instance labels. Finer annotations, such as trunk, branch, or detailed foliage labels, would require prior customization of tree assets with material properties that may not be available in Unreal Engine gaming assets. To partially mitigate this limitation, we have extended the dataset to include leaf-wood semantic labels (Fig. 10 in Section A.8). This represents the most fine-grained annotation achievable with most existing tree assets designed for gaming applications. In particular, leaf–wood separation enables a key ecological application (Disney et al., 2018): fitting Quantitative Structure Models (QSMs) from wood points of individual trees to study branching patterns and forest growth dynamics.

### Benchmark dataset from real world

Models trained on our synthetic dataset needs to be validated on the real-world dataset to study the effectiveness of our approach. FOR-Instance (Puliti et al., 2023) is a recently published UAV laser scan dataset that has previously been used for tree segmentation development and evaluation (Straker et al., 2023; Wielgosz et al., 2024; Xiang et al., 2024), making it a suitable choice for this study.

**Fig. 5:**
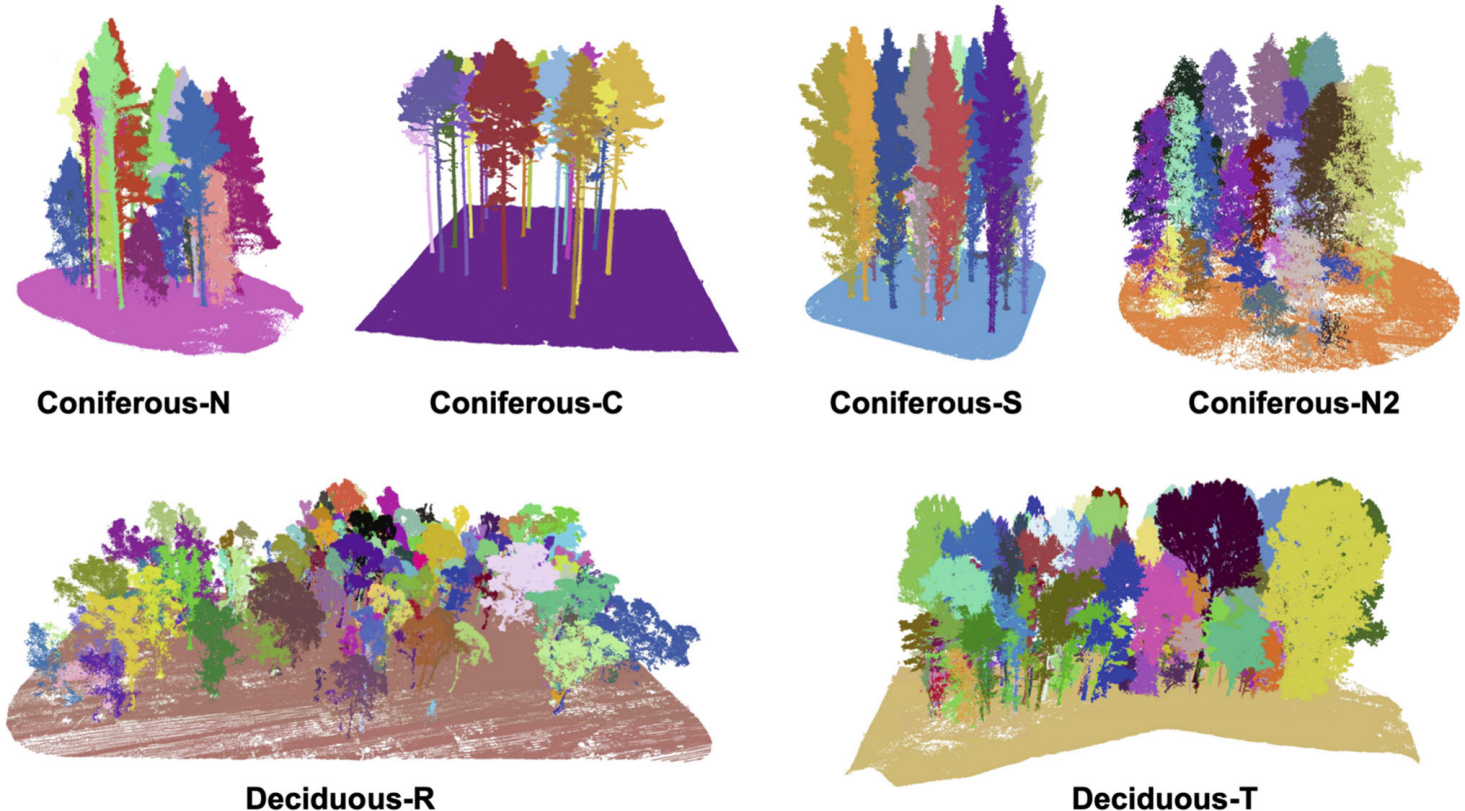
Annotating the FOR-Instance dataset (Puliti et al., 2023) took two skilled annotators six months, in addition to the time spent on data collection. It covers 3 coniferous scenes and 2 deciduous scenes totalling 2.79 hectares, and Coniferous-N2 is a supplementary dataset from Xiang et al. (2024). Annotated plots from each region are illustrated

The FOR-Instance data set covers 5 forest regions (Table 3), including 3 coniferous regions (Coniferous-N, Coniferous-S, Coniferous-S) and 2 deciduous regions (Deciduous-R, Deciduous-T), totalling 2.79 hectares of forest plots (Fig. 5). These forest plots are collected mainly from Norway, Central Europe, Australia, and New Zealand. However, most of the forest plots consist of coniferous pine trees from the Coniferous-N region, while the rest of the region consists only of 1 or 2 forest plots. Scanning and annotating forest plots is costly, particularly for the complex deciduous forests.

FOR-Instance contains both instance and semantic segmentation labels. Each tree in the point clouds has a unique segmentation ID, similar to our synthetic dataset. Semantic labels cover five classes: stem, woody branches, live branches, low-vegetation, and ground. As mentioned before, generating the full 5-class labels would require customising 3D tree assets with distinct material properties, which is not readily available in the high-quality gaming assets we use. For consistency therefore, we remapped the original 5 semantic labels of the FOR-Instance dataset into the binary classes (tree: woody branches, live branches, low-vegetation. non-tree: low-vegetation, ground). Our synthetic data collection method produces forest plots with point densities exceeding 1000 $points/m^2$, consistent with those in the FOR-Instance data set collected by UAV laser scanning.

### Tree segmentation algorithm

**Fig. 6:**
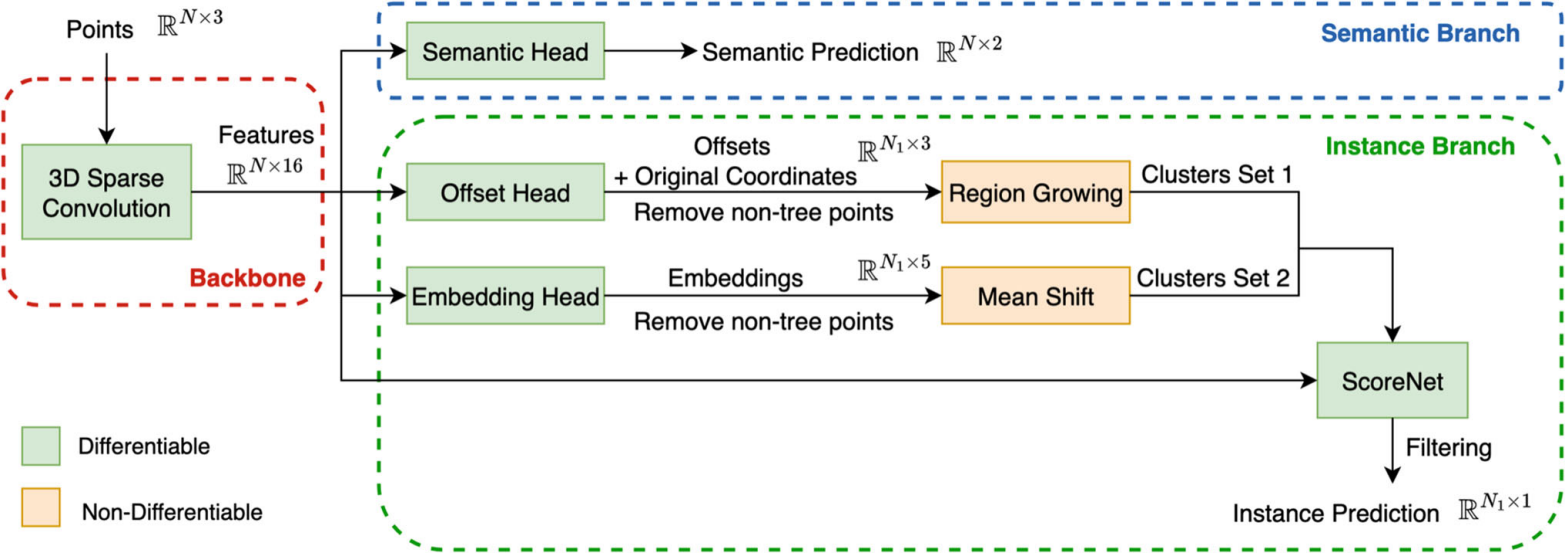
The panoptic tree segmentation algorithm (Xiang et al., 2024) $\mathcal {A}$ adapted for this study. Panoptic segmentation addresses instance and semantic segmentation simultaneously. $\mathcal {A}$ uses the Minkowski Engine, a 3D CNN, as its backbone followed by semantic and instance branches. In our experiments, we simplify semantic prediction from five classes to binary: tree and non-tree

For the tree segmentation algorithm, we use ForAINet from Xiang et al. (2024) (denoted by $\mathcal {A}$), which was developed using the FOR-Instance dataset and considered the state-of-the-art algorithm for instance and semantic segmentation of forest point clouds (Fig. 6).

In summary, $\mathcal {A}$ is adapted from the PointGroup algorithm (Jiang et al., 2020) and uses Minkowski Engine (Choy et al., 2019), a U-Net for sparse 3D convolutions in point clouds, as its backbone. The backbone is followed by three heads. The semantic head takes backbone features at each point and classifies them into the five semantic classes. Two other heads that are complementary for the instance segmentation:

1. an embedding head that embeds the backbone feature into a 5 dimensional space and then performs a clustering with mean-shift in this 5D space to detect tree clusters from embeddings;
2. an offset head that shifts the points in the original 3D space via predicted 3D offsets, followed by another clustering with region-growing to detect tree clusters.

Finally, the two instance segmentation heads are followed by a small neural network module ScoreNet (Jiang et al., 2020) that learns to score each detected cluster by comparing ground truth IoU with predicted IoU. During inference, these scores, together with the detected clusters, are passed to Non-Maximal Suppression to obtain the final predictions of instance segmentation.

To train the model, 8 m radius cylinders are randomly sampled from the forest plots as individual data samples. In addition to the basic training setting, Xiang et al. (2024) applied a data augmentation strategy called ‘TreeMix3D’, which they found to boost model performance. This augmentation increases forest structure diversity by randomly replacing 30% of the trees from one cylinder sample with trees from another cylinder sample. During inference, the model use grid cylinder sampling, placing cylinders systematically to cover the plot; each is inferred independently, with results optionally merged for plot-level inference. The 8 m radius cylinders are designed mainly for tree sizes commonly seen in deciduous and coniferous plots. For consistency, we exclude the RedWood scene from model training, since the large tree sizes makes them unsuitable for this segmentation algorithm.

Our experiments studying the effectiveness of synthetic data for instance segmentation, use a simplified form $\mathcal {A}_{\text {simp}}$ of the original algorithm which we now denote $\mathcal {A}_{\text {full}}$:

- The semantic head uses just 2 binary classes, as already explained.
- Instead of jointly training ScoreNet with other modules from very early epochs, we first train modules and then save the detected clusters to train ScoreNet separately. Costly non-differentiable clustering is then done just once, after other modules have been fully trained, significantly speeding up training on large-scale synthetic data.

### Experiments

We first pre-train $\mathcal {A}_{\text {simp}}$ on the full-scale synthetic Sim_11,187_ data for 150 epochs, where ‘Sim’ denotes synthetic data, ‘Real’ denotes real FOR-Instance data, the first subscript indicates the number of scenes, and the second the number of plots. Sim_11,187_ consists of 11 scenes, totalling 187 plots and 13635 cylinder samples (see also Table 6 for scene compositions). This pre-training should provide a strong feature extraction network benefiting from the diversity of the synthetic data that includes coniferous, deciduous, and rainforest scenes. The pretrained model is then fine-tuned on the corresponding coniferous and deciduous forest plots from the real FOR-Instance dataset. Unless otherwise specified, we report metrics as the mean over all cylinder samples obtained during inference, each with its own accuracy, recall, and F-score.

We have built two few shot datasets, subsets of FOR-Instance with few labelled plots to assess model performance with limited data (Table 4):

- Real-C_3,3_, contains the 3 coniferous scenes from the original FOR-Instance dataset, one plot from each coniferous region (Real-C-N_1,1_, Real-C-C_1,1_, and Real-C-S_1,1_).
- Real-D_2,2_, contains the 2 deciduous scenes from FOR-Instance dataset, one plot from each deciduous region (Real-D-R_1,1_ and Real-D-T_1,1_). (These are the only deciduous plots available in the FOR-Instance dataset due to the difficulty in scanning and annotating deciduous forests.)

**Table 3:** Overview of the FOR-Instance data set from Puliti et al. (2023). NIBIO2 is not part of the FOR-Instance dataset but is included in the training set in Xiang et al. (2024). Note that RMIT and TUWIEN are deciduous forests and the rest are coniferous forests. Except for NIBIO and NIBIO2, the rest of the regions only have 1 or 2 training plots available

Scene Name	Original Name	Tree Species	Plots			Point Density (points/m^2^)
			Train	Val	Test	
Coniferous-N	NIBIO	Norway Spruce (dominated)	8	6	6	9529
Coniferous-C	CULS	Scots Pine	1	1	1	2585
Coniferous-S	SCION	Monterey Pine	2	1	2	4576
Deciduous-R	RMIT	Eucalyptus pulchella	1	0	1	498
Deciduous-T	TUWIEN	Deciduous species in Austria	1	0	1	1717
Coniferous-N2	NIBIO2	Norway Spruce (dominated)	29	6	15	>1000

**Table 4:** Real datasets used in few shot learning experiments. The subscripts denote the number of scenes and the number of plots. Besides the full dataset Real_6,42_, we organised few-shot subsets for coniferous (Real-C) and deciduous forests (Real-D). We report cylinder sample counts from each subset and their percentages of the full dataset

Dataset	Scenes	Plots	Samples	Percentage (%)
Real_6,42_	Coniferous-N, Coniferous-C, Coniferous-S, Coniferous-N2	42	1051	100
	Deciduous-R, Deciduous-T			
Real-C_3,3_	Coniferous-N, Coniferous-C, Coniferous-S	3	76	7.2
Real-C-N_1,1_	Coniferous-N	1	32	3.1
Real-C-C_1,1_	Coniferous-C	1	31	2.9
Real-C-S_1,1_	Coniferous-S	1	23	2.2
Real-D_2,2_	Deciduous-R, Deciduous-T	2	160	15.2
Real-D-R_1,1_	Deciduous-R	1	72	6.8
Real-D-T_1,1_	Deciduous-T	1	88	8.4

**Table 5:** Pretraining on synthetic data, and using minimal real data fine-tuning, performs competitively with a much larger, labelled, real dataset. F1 scores improve significantly for small, labelled coniferous regions when synthetic (mixed deciduous/coniferous) data is available for pre-training. Deciduous plots show a similar pattern, with the same pretraining data

Test	Pretrain	Train/Tune	Experiment	Real Data Used (%)	F1-score (%)
Coniferous-N	-	Real_6,42_	Training on all real plots from scratch	100	79.8
	-	Real-C_3,3_	Training on 3 coniferous plots from scratch	7.2	50.5
	Sim_11,187_	-	Zero-shot transfer from synthetic plots	0.0	59.4
	Sim_11,187_	Real-C_3,3_	Fine-tuning on 3 coniferous plots	7.2	79.4
	Sim_11,187_	Real-C-N_1,1_	Fine-tuning on 1 plot from Coniferous-N	3.1	77.1
Deciduous-R	-	Real_6,42_	Training on all real plots from scratch	100	69.9
	-	Real-D_2,2_	Training on 2 deciduous plots from scratch	15.2	31.6
	Sim_11,187_	-	Zero-shot transfer from synthetic plots	0.0	61.7
	Sim_11,187_	Real-D_2,2_	Fine-tuning on 2 deciduous plots	15.2	70.0
	Sim_11,187_	Real-D-R_1,1_	Fine-tuning on 1 plot from Deciduous-R	6.8	69.3

Real-C_3,3_ only accounts for 7.2% of the total available samples, and for Real-D_2,2_ it is 15.2% (Table 4). We fine-tune pretrained models on each few-shot dataset and compare them to models trained from scratch. This comparison will show how much $\mathcal {A}_{\text {simp}}$ benefits from pre-training on synthetic data versus training solely on limited real data, crucial for effective few-shot learning. We also try fine-tuning the pretrained model on a single real plot of each individual forest region from the few-shot datasets. This is an even more extreme case of few-shot. It is also a practical scenario for a forest manager with only a single annotated forest plot available, and happens in the FOR-Instance dataset, where most regions, except Coniferous-N and Coniferous-N2, have just 1 or 2 plots.

We also compare our few-shot learning results with the results obtained from training with $\mathcal {A}_{\text {simp}}$ on the full-scale FOR-Instance dataset Real_6,42_, which contains 6 scenes, totalling 42 plots and 1051 samples (Table 4), to see if we can obtain competitive results with only a few forest plots.

### Implementation Details

Pretraining on the synthetic dataset Sim_11,187_ totals 150 epochs. For fine-tuning, the pretrained models are fine-tuned on real datasets for 60 epochs. Baselines are built by training the models from scratch on the real datasets for 120 epochs. All models are trained with the data augmentation setting ‘TreeMix3D’ as discussed in Section 3.4, which is in line with Xiang et al. (2024). ScoreNet is not initially included in training because the point clustering required would substantially slow down the process. Instead, once other layers are trained, detected clusters are saved and used to train ScoreNet separately (for 30 epochs) to score and filter the clusters. The initial learning rate for pretraining is 0.001, and the initial learning rate for fine-tuning is 0.0001. The batch size is 8. All models are trained on a NVIDIA GeForce RTX 4080 GPU, with 128G RAM.

## Results

### Synthetic data can enhance instance segmentation with minimal use of real plots

In Table 5, we compare the performance of $\mathcal {A}_{\text {simp}}$ trained in different settings in coniferous and deciduous plots. More detail can be found in Table 10 and Table 11 in Section A.3.

**Fig. 7:**
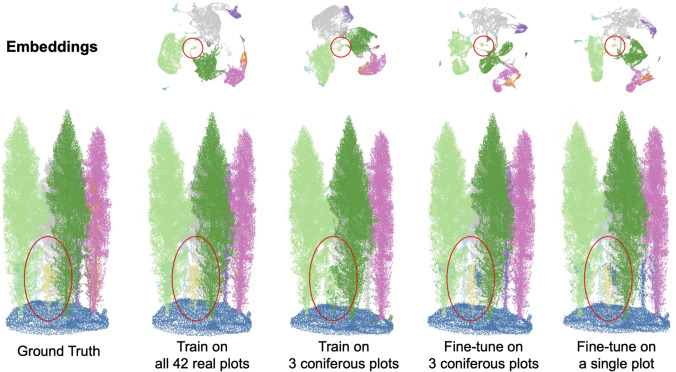
Pretraining on synthetic data enables the algorithm to learn representations like those from real data, with fine-tuning on just a single forest plot. We present a sample from Coniferous-N, where embeddings and points shifted by offsets serve as the foundation, for instance segmentation. Both embeddings and points are colourized with ground-truth labels and projected in 2D using UMAP (Mcinnes et al., 2018). Training only on three coniferous plots, the algorithm struggles to learn disentangled representations, as seen in segmentation results where small trees in the understory are not segmented, noted within the red circle

Training from scratch with the reduced coniferous data set Real-C_3,3_ leads to a significant drop in F1 score of more than 29%. Pretraining with synthetic data and then fine-tuning with Real-C_3,3_ fully restores the drop in performance (In fact this improvement is evident in all coniferous regions: see Table 10.) Even extreme few-shot training, using only 3.1% of the samples, is competitive, dropping just a couple of percentage points on F1. Fig. 7 shows learnt representations and segmentation results for a Coniferous-N sample in various settings; synthetic data pretraining facilitates the learning of comparable representations, even with minimal fine-tuning.

A similar pattern of improvement can be observed in Deciduous-R (Table 5) and other deciduous plots (Table 11). Training only on Real-D_2,2_ reduces the F1 score significantly compared to the full-scale Real_6,42_. For Deciduous-R, it drops from 69.9% to 31.6%, despite Real-D_2,2_ covering all available plots. Fine-tuning the pretrained model on Real-D_2,2_ fully restores the loss.

Zero-shot transfer from the model pretrained only on synthetic data (Sim_11,187_) outperforms training from scratch on the few-shot real datasets Real-C_3,3_ and Real-D_2,2_. On Deciduous-R, its zero-shot F1 of 61.7% is over 30 points higher than training from scratch on Real-D_2,2_ (31.6% vs. 61.7%). Fine-tuning on Real-D_2,2_ further increases F1 to 70.0%, but the strong zero-shot performance on unseen forest plots highlights the generalisation capacity of our synthetic dataset and helps explain the earlier few-shot gains.

In terms of computational efficiency, fine-tuning the model needs only 60 epochs, compared to 120 epochs when training from scratch. Our synthetic data approach to pre-training achieves competitive segmentation performance, with substantial savings both of annotation effort and computational cost.

### Key elements contributing to the effectiveness of synthetic data

**Table 6:** Synthetic datasets in our ablation study analyze three factors: simulation physics (Nodal_4,20_ vs. Sim_4,20_), scene diversity (Sim_4,20_ vs. Sim_11,55_), and dataset scale (Sim_11,55_ vs. Sim_11,187_). Nodal points derive from tree mesh nodes, while Sim points come from LiDAR simulations. Both Sim_4,20_ and Sim_11,55_ cover 30% of available plots (5 plots per scene) from their scenes

Dataset	Scenes	Plots	LiDAR
Nodal_4,20_	Deciduous1, Deciduous2, Deciduous3, Rainforest	20	✗
Sim_4,20_	Deciduous1, Deciduous2, Deciduous3, Rainforest	20	✓
Sim_11,55_	Deciduous1, Deciduous2, Deciduous3, Deciduous4, Deciduous5, Deciduous6,	55	✓
	Coniferous1, Coniferous2, Coniferous3, Coniferous4, Rainforest		
Sim_11,187_	Deciduous1, Deciduous2, Deciduous3, Deciduous4, Deciduous5, Deciduous6,	187	✓
	Coniferous1, Coniferous2, Coniferous3, Coniferous4, Rainforest		

**Fig. 8:**
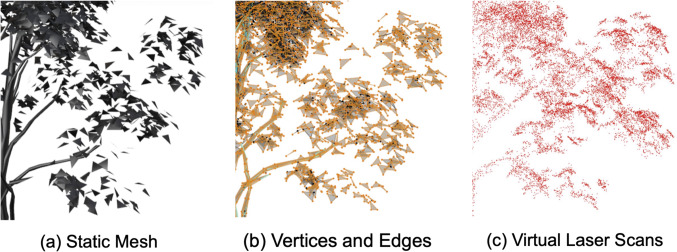
Nodal points from tree meshes provide a simple way to obtain point clouds. A large ash tree model from the Deciduous3 scene is shown. A rendered tree model consists of textures and a static mesh. The static mesh, made of vertices and edges, directly yields nodal points as point clouds. LiDAR simulation point clouds are based on UAV laser scans and are denser than these nodal points

In Section 1, we point out three key strengths of our synthetic data generation pipeline: diverse forest scenes, large-scale forest generation, and physics-based laser scans for point generation. We perform ablation studies to study the role of each of the three (Table 6). We pre-train $\mathcal {A}_{\text {simp}}$ on each of these synthetic datasets in the same setting of pre-training on Sim_11,187_, as described in Section 3.6. Then the zero-shot learning experiments directly evaluate the model obtained in each forest region from the real datasets.

We use four forest scenes from the SPREAD dataset (Feng et al., 2025), each with 5 plots, totalling 20 plots. The data set incorporates tree mesh points that arguably approximate point clouds (Fig. 8). We refer to the point clouds from tree mesh points as Nodal_4,20_, and those from UAV-LiDAR simulation with HELIOS as Sim_4,20_ (Table 6).

Table 7 shows the results for Coniferous-C. More detail can be found in Table 12 in Section A.4. Training with UAV-LiDAR simulation (Sim_4,20_) significantly enhances performance, whereas nodal point clouds from tree mesh (Nodal_4,20_) performs poorly: F1 score in Table 7 improved from 5.4% to 42.6%. A similar pattern of improvement can be observed for other regions (Table 12 in Section A.4). Note that HELIOS ray-tracing simulates UAV scanning, producing over 1000 points/m^2^, unlike tree meshes, which average 100-200 points/m^2^ and have different distributions with denser canopy points from laser scans.

We extend Sim_4,20_ to Sim_11,55_ to study scene diversity. Sim_11,20_ adds three deciduous and four coniferous scenes not in Sim_4,20_ and Sim_4,20_, with each scene having 5 plots like Sim_4,20_. There is consistent improvement across all regions (Table 12), with Coniferous-C’s F-1 score increasing by 38.9% from 42.6% to 81.5%.

We use the full-scale synthetic data set Sim_11,187_ to study the effects of the size of the data set. Compared with Sim_11,55_, it includes all available plots (17 per forest scene) for the 11 scenes. More training data helps the model learn scene variations and enhances F1 performance from 81.5% to 87%.

**Fig. 9:**
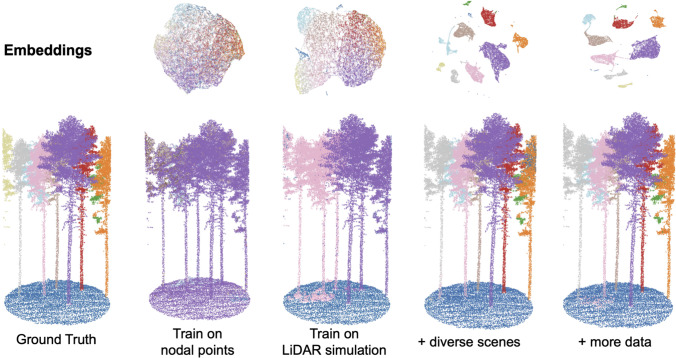
Synthetic data helps the algorithm learn disentangled representations. A Coniferous-C data sample with embeddings and offset-shifted points, is colorized with ground truth labels and projected into 2D using UMAP (Mcinnes et al., 2018). Compared with training at nodal points, disentanglement is enhanced as we add: LiDAR simulation; diverse scenes, especially newly included coniferous ones; more simulated data

**Fig. 10:**
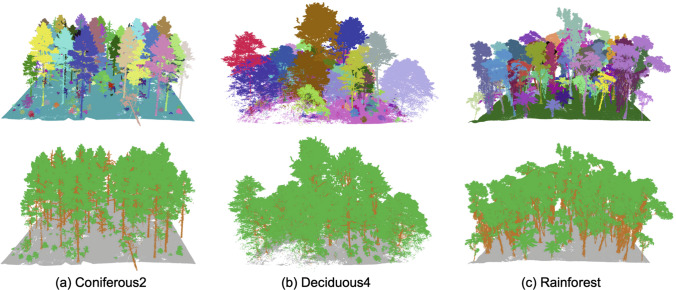
Extended dataset with leaf–wood labels enabling quantitative analysis of forest structure. Instance labels (top) and semantic labels (bottom) for ground, leaf, and wood, which are typically labor-intensive to generate through manual annotation

To summarize, the algorithm effectively learns about forest structures from synthetic data, crucial for real-world applicability — see few-shot studies in Section 4.1. Enhancing scene diversity seems to offer greater benefits than simply expanding the size of the dataset presumably by playing a key role in learning disentangled representations (Fig. 9).

### Bridging domain gaps with mixed training of synthetic and real data

In Section 4.1, we conduct few-shot experiments with $\mathcal {A}_{\text {simp}}$ to show the effectiveness of the synthetic data. However, pretraining only on synthetic data may not produce representations as strong or generalisable as those learnt from a combination of synthetic and real data. Furthermore, the original real data includes five semantic labels for the model to learn, representing not only a practical application, but also an enriched multi-task learning scenario that could improve algorithm performance.

We consider the synthetic data as the source domain ($\mathcal {S}$) and the real data as the target domain ($\mathcal {T}$). In practice, $\mathcal {T}$ can be unlabelled ($\mathcal {T}_{\text {unlabel}}$), as is common in forest surveys, or labelled ($\mathcal {T}_{\text {label}}$), as in the FOR-Instance dataset. In this section, we focus on the labelled setting to examine whether pretraining in a combination of $\mathcal {S}$ and $\mathcal {T}_{\text {label}}$, including its full 5-class semantic labels, can produce stronger performance. To investigate this, we adopt the original algorithm $\mathcal {A}_{\text {full}}$ in a mixed training set.

In mixed training, we use datasets from both $\mathcal {S}$ and $\mathcal {T}_{\text {label}}$. Although $\mathcal {A}_{\text {full}}$ is designed for five semantic classes, $\mathcal {S}$ provides only binary semantic labels (tree / non-tree). To accommodate both, the semantic branch is modified to have two heads: one predicts five classes for $\mathcal {T}_{\text {label}}$, and the other predicts binary labels for $\mathcal {S}$. The instance branch remains unchanged. Each batch contains an equal number of synthetic and real samples. After pretraining, we continue to fine-tune the model on only the real data set from $\mathcal {T}_{\text {label}}$, improving alignment with the real data. We pretrain on the mixed dataset for 120 epochs and fine-tuned solely on the real dataset for 30 epochs. To accelerate training, $\mathcal {A}_{\text {full}}$ is initialized with the pretrained weights of Sim_11,187_, acquired in previous experiments.

In Table 8, mixed-training F1 scores are broadly consistent with those reported by Xiang et al. (2024) except for the Deciduous-T plot, where both our mixed training and our reproduction of their official model produce substantially lower results. As detailed in Section A.6, this discrepancy largely stems from F1 sensitivity to the IoU matching threshold τ, not model quality. Deciduous-T contains large tree crowns that span multiple cylinder samples (radius: 8 m), producing fragmented predictions whose IoU against the GT falls just below the default τ=0.5. An ablation over τ confirms this: reducing τ to 0.35 recovers an F1 of 68.5%, close to the reported 69.4%, accounting for most of the discrepancy. The remaining gap may reflect undocumented implementation details in the original code that could not be fully controlled in our reproduction. This further supports our choice in the few-shot experiments to report F1 as a direct mean over cylinder-level predictions without plot-level aggregation, which more faithfully reflects representation quality.

**Table 7:** Three factors enhance the value of synthetic data for training: physics-based laser scans, scene diversity, and increased data. Adding physics-based LiDAR simulation (Sim_4,20_) improves F1 score over using tree mesh nodal points (Nodal_4,20_). Greater scene diversity further boosts performance (Sim_11,55_ versus Sim_4,20_), with additional data providing a smaller gain (Sim_11,187_ versus Sim_11,55_)

Test	Train	Ablation Experiment	F1-score (%)
Coniferous-C	Nodal_4,20_	Nodal points from 4 scenes (5 plots per scene)	5.4
	Sim_4,20_	Simulated LiDAR from the same 4 scenes (5 plots per scene)	42.6
	Sim_11,55_	+7 additional scenes (total 11 scenes, 5 plots each)	81.5
	Sim_11,187_	+More plots per scene (total 11 scenes, 17 plots each)	87.0

Overall, Table 8 shows that mixed training matches state-of-the-art performance but does not surpass it. A likely explanation is that the test plots are drawn from the same forest regions as $\mathcal {T}_{\text {label}}$, limiting the scope for synthetic data to provide additional benefit. To test this hypothesis, we perform mixed training experiments in a domain shift setting (Section A.5), where the model is trained on a reduced $\mathcal {T}_{\text {label}}$ excluding one forest type (coniferous or deciduous) and evaluated on the other. In this setting, adding synthetic data consistently mitigates the domain gap and significantly improves F1 scores – on Coniferous-C and Deciduous-R, mixed training achieves F1 scores of 100% and 66.7%, respectively, exceeding or matching state-of-the-art performance. This confirms that the diversity and scale of our synthetic data enables the model to bridge domain gaps more effectively, particularly when a distribution shift exists between training and test sets. Further suggestions are provided in Section 5.2.

## Discussion

### Improving the reusability of real-world labelled scans with procedural foliage generation

High-quality field scans remain significant for our synthetic data pipeline, as they provide the detailed, labelled laser scans of individual trees needed to create realistic 3D tree assets. Traditionally, new study areas require scanning entire forest plots and manually annotating each tree to produce training data, a process that is time-consuming and costly (Calders et al., 2022; Puliti et al., 2023; Weiser et al., 2022).

The procedural foliage generation ($\mathcal {A}_{\text {PFG}}$) offers a more efficient alternative. Once a library of 3D tree assets has been created from existing field scans, $\mathcal {A}_{\text {PFG}}$ can recombine these assets to generate new forest plots customised to a given region of interest. This allows researchers to produce large, labelled datasets for new areas without rescanning or re-annotating whole plots, greatly improving the reusability of field-collected data.

For example, the scene ‘Deciduous3’ in our synthetic dataset is a 250 m × 250 m (6.25 ha) forest containing 2,053 trees (328 trees/ha). These are generated from 17 scanned tree meshes (two species: Ash and Linden), scaled, rotated, and placed under procedural rules to represent different canopy levels (Table 16). Even though this procedurally generated scene has limited species diversity, it can still capture the structural variability needed for effective model training, offering a practical balance between ecological realism and machine learning efficiency.

**Table 8:** Our model trained on mixed data achieves results comparable to state-of-the-art, without exceeding it. F1-scores (%) for mixed training, results in Xiang et al. (2024), and our reproduction of Xiang et al. (2024), are similar across regions. Deciduous-T is an exception where both our result and the reproduction are notably lower than reported 69.4% by the authors. Metrics are computed at the plot level by aggregating predictions from all cylinder samples within each plot, following the same procedure of Xiang et al. (2024) to ensure comparability

Test Set	Mixed Training	Reproduced
Coniferous-N	92.0	91.6
Coniferous-C	90.5	93.0
Coniferous-S	92.9	89.4
Deciduous-R	66.1	67.2
Deciduous-T	47.4	46.6

This sampling approach can be applied to a specific region of interest and is compatible with our synthetic data generation pipeline. In fact, some laser scan data sets, such as the FOR-Species20K dataset (Puliti et al., 2024), have recently been made available to derive such individual tree meshes. Alternatively, high-fidelity reconstruction techniques such as 3D Gaussian Splatting (Kerbl et al., 2023) can be experimented with to acquire high-fidelity tree assets with RGB cameras. In either approach, sampling individual trees for procedural foliage generation would be more efficient and reusable than traditional field work. The generated tree assets will be compatible with the data generation pipeline as presented in this paper. In addition, the generation of labels for machine learning would be cost-efficient.

### Further work

We provide some recommendations for future research aimed at developing robust representations for forest vision tasks using our synthetic data pipeline and the full-scale synthetic dataset generated via LiDAR simulation (Sim_11,187_).

**Mixed training with synthetic data when the target domain has no labelled data available ** (Dong et al., 2024; Hatano et al., 2024). In addition to the scenario of Section 4.3, in the pretraining phase, the standard supervision loss can be applied to $\mathcal {S}$ to learn task-specific features, for example segmentation. We then propose to incorporate a domain adaptation loss, such as those used in prior work on multimodal video data (Dong et al., 2024) to align representations between $\mathcal {S}$ and $\mathcal {T}_{\text {unlabel}}$. In that way, the pretrained representation can be better aligned to a specific region of interest.

**Scaling to neural architectures of higher capacity** is another important aspect to consider. Although our study uses the algorithm from Xiang et al. (2024) with its 3D convolutional neural network as backbone, Transformer architectures have shown promising results in point cloud processing (Wu et al., 2024) and have recently been successfully applied to tree segmentation tasks (Xiang et al., 2025). Empirical scaling laws for deep learning (Zhai et al., 2022) show that Transformer architectures often continue to improve with increasing quantities of data, without clear saturation. Such models could especially benefit from using our synthetic data learn even stronger representations.

**Expanding synthetic data generation** using our CAMP3D pipeline. For forest scenes from Unreal Engine, physics-based LiDAR simulation can be applied to to not only UAV laser scans but also terrestrial, mobile, and airborne laser scans. Scene diversity can be enhanced by creating region-specific scenes, as noted in Section 5.1. Unlimited variations of forest scenes can be generated by altering random seeds in procedural foliage generation. Currently, our experiments run with only two semantic labels, compared to five in the real dataset, which leads to some performance loss. This is a limitation of our generated data, and so is the best that is currently possible with few shot learning. Future work could explore generating finer semantic labels from Unreal Engine’s tree assets to assess potential performance improvements.

We have open-sourced our data pipeline to allow the community to enhance and contribute new forest scenes and laser scans, aiding in the advancement of forest vision systems.

**Evaluation benchmark:** In addition to model training, our synthetic data could serve as a benchmark to evaluate the performance of different tree segmentation algorithms. This is possible because of its more extensive and balanced coverage across forest types.

## Conclusion

We have introduced CAMP3D, a novel synthetic data generation pipeline for 3D forest vision to address a key challenge in this domain— the scarcity of labelled data for supervised machine learning. Procedural foliage generation originated in the game industry but is rooted in ecological theories and that may partly explain its effectiveness. Using extensive tree assets from game engines, we can generate large-scale, diverse forest scenes. Combined with advanced LiDAR simulation, this enables a fully automated data generation pipeline to create machine learning-ready forest datasets. This facility supports a compelling methodology for more efficient field campaigns.

We used the pipeline to create an extensive 3D forest dataset that covers 12 scenes and 75 hectares, exceeding the size of any real dataset in this field that we have encountered so far. Comprehensive experiments confirmed the efficacy of the synthetic dataset for learning representations for instance segmentation. We have also highlighted three key elements that underpin the success of synthetic data: physics-based data generation, scene diversity, and a large scale dataset. We hope and expect that researchers will be able to take advantage of our data pipeline and generated dataset to further advance forest vision using synthetic data.

## Data Availability

The synthetic dataset generated and used in this study is publicly available at https://zenodo.org/records/17106960. The CAMP3D data generation pipeline is available at https://github.com/yihshe/CAMP3D.git.

## Appendix

### LiDAR simulation details

**Table 9:** Our virtual survey resembles a real world UAV mission. The RIEGL VUX-1 UAV scanner is widely used. For Redwood scene we set the Relative Altitude as 120 *m* because of the tree’s large size. All parameters are customizable in our data pipeline

Parameter	Value
Scanner Model	RIEGL VUX-1 UAV
Pulse Frequency	100 kHz
Max Returns (100 kHz)	15
Flight Pattern	Criss-cross
Flight Spacing	20.0 m
Flight Speed	5.0 m/s
Relative Altitude	60.0 m

**Table 10:** The algorithm pretrained on our synthetic data can achieve competitive performance, with fine-tuning only on a few real forest plots. This table presents the results for coniferous regions. The improvement of F1 score is significant compared to training on these few-shot dataset from scratch, indicating that our synthetic data contains useful prior knowledge for the algorithm to learn in order to generalise. All values are in percentages (%)

Test	Pretrain	Train/Tune	Mean IoU	Precision	Recall	F1-score
Coniferous-N	-	Real_6,42_	68.7	91.4	73.6	79.8
	-	Real-C_3,3_	39.2	80.2	40.2	50.5
	Sim_11,187_	-	50.3	71.5	56.8	59.4
	Sim_11,187_	Real-C_3,3_	67.1	94.8	71.1	79.4
	Sim_11,187_	Real-C-N_1,1_	66.0	91.3	69.4	77.1
Coniferous-C	-	Real_6,42_	88.3	95.1	93.0	93.4
	-	Real-C_3,3_	59.7	98.5	68.7	79.5
	Sim_11,187_	-	81.2	96.6	81.6	87.0
	Sim_11,187_	Real-C_3,3_	79.5	99.5	81.6	88.1
	Sim_11,187_	Real-C-C_1,1_	78.0	99.3	79.6	87.1
Coniferous-S	-	Real_6,42_	68.6	87.1	75.2	78.9
	-	Real-C_3,3_	54.4	91.4	59.1	69.2
	Sim_11,187_	-	51.9	59.9	55.8	55.9
	Sim_11,187_	Real-C_3,3_	67.4	91.7	75.4	81.4
	Sim_11,187_	Real-C-S_1,1_	61.1	92.7	66.9	75.7

HELIOS generates labels by sampling attributes from the 3D objects used in the simulation. For mesh-based objects, the simulator directly uses the attributes defined in the meshes rather than voxelizing them. During simulation, rays are cast through the scene, and the intersections with object surfaces determine the point locations. The corresponding object attributes are then recorded as point labels. To enable instance-level labeling, we export individual trees from Unreal Engine to Blender and assign each object a unique ID attribute. A Blender add-on developed by Neumann et al. (2022) is integrated into our pipeline to convert forest scenes into a data structure readable by the simulator.

Based on the customised forest, our pipeline will generate a survey file for each scene that will be used by HELIOS to conduct the survey. Our virtual survey resembles how UAV-laser scans work in the real world. Table 9 gives an overview of the key parameters of the survey. We use RIEGL VUX-1 UAV—a popular scanner model in real-world forest surveys—as our scanner. Criss-cross is used as the flight pattern, which, compared to the Parallel option, yields denser point clouds. The Pulse Frequency (100 kHz) and Flight Speed (5 m/s) jointly determine the sampling density along the track in our simulated survey. The chosen values match the typical RIEGL VUX-1 UAV survey settings in forest applications (Puliti et al., 2023). Relative Altitude is set at 60.0 m, which will work for all forest scenes except the RedWood scene, where it is set at 120 m given the size of the large tree.

With the forest scenes and survey files ready, the pipeline will proceed with the LiDAR simulation to generate UAV-laser scans with the desired annotations.

**Table 11:** The algorithm pretrained on our synthetic data can achieve competitive performance in deciduous regions, with fine-tuning on only a few real forest plots. The pattern of improvement is similar to coniferous plots. These results strongly suggest that our synthetic data contain prior knowledge that can make the algorithm generalise for different forest types. All values are in percentages (%)

Test Set	Pretrain	Train/Tune	Mean IoU	Precision	Recall	F1-score
Deciduous-R	-	Real_6,42_	56.0	86.6	60.9	69.9
	-	Real-D_2,2_	27.1	67.1	22.4	31.6
	Sim_11,187_	-	54.8	74.5	55.6	61.7
	Sim_11,187_	Real-D_2,2_	61.5	81.7	63.7	70.0
	Sim_11,187_	Real-D-R_1,1_	61.8	82.1	62.9	69.3
Deciduous-T	-	Real_6,42_	48.3	63.8	48.8	52.5
	-	Real-D_2,2_	29.9	52.1	30.7	32.7
	Sim_11,187_	-	49.4	49.8	52.8	45.7
	Sim_11,187_	Real-D_2,2_	54.6	73.8	58.0	59.9
	Sim_11,187_	Real-D-T_1,1_	49.0	76.4	49.4	54.9

**Table 12:** Three factors plays significant roles in generating strong synthetic data from our pipeline, as revealed in the zero-shot experiment. (1) Physics-based simulation, by comparing improvement of F1-score from the algorithm trained on LiDAR simulation (Sim_4,20_) against nodal points from tree meshes Nodal_4,20_. (2) More diverse scenes, by comparing the improvement from training on Sim_4,20_ against Sim_11,55_. Both datasets have five plots for each scene. (3) More data, by comparing the learning outcome of Sim_11,187_ and Sim_11,55_. Both have the same number of scenes. All values in percentages (%)

Test	Train	Ablation Factor	Mean IoU	Precision	Recall	F1-score
Coniferous-N	Nodal_4,20_	nodal points	12.5	4.4	11.4	6.0
	Sim_4,20_	LiDAR simulation	29.0	37.0	28.6	28.4
	Sim_11,55_	+ diverse scenes	44.9	69.0	45.1	50.8
	Sim_11,187_	+ more data	50.3	71.5	56.8	59.4
Coniferous-C	Nodal_4,20_	nodal points	20.7	3.5	15.5	5.4
	Sim_4,20_	LiDAR simulation	38.6	63.5	34.3	42.6
	Sim_11,55_	+ diverse scenes	72.4	95.7	74.0	81.5
	Sim_11,187_	+ more data	81.2	96.6	81.6	87.0
Coniferous-S	Nodal_4,20_	nodal points	28.5	11.7	21.0	14.0
	Sim_4,20_	LiDAR simulation	44.0	70.7	46.2	52.4
	Sim_11,55_	+ diverse scenes	46.2	63.7	49.9	53.0
	Sim_11,187_	+ more data	51.9	59.9	55.8	55.9
Deciduous-R	Nodal_4,20_	nodal points	16.9	2.9	5.8	2.7
	Sim_4,20_	LiDAR simulation	34.7	44.2	32.5	34.8
	Sim_11,55_	+ diverse scenes	50.4	66.1	51.1	54.5
	Sim_11,187_	+ more data	54.8	74.5	55.6	61.7
Deciduous-T	Nodal_4,20_	nodal points	20.1	3.7	16.8	5.2
	Sim_4,20_	LiDAR simulation	39.2	26.5	42.6	29.0
	Sim_11,55_	+ diverse scenes	45.7	40.9	49.3	40.6
	Sim_11,187_	+ more data	49.4	49.8	52.8	45.7

**Table 13:** Mixed training with synthetic data substantially improves model performance under domain shift between real training and test data. Metrics are aggregated at the plot level. Models trained only on deciduous plots but tested on unseen coniferous plots achieve much higher F1 when synthetic data are added to training. A similar gain is observed for models trained on coniferous plots and tested on unseen deciduous plots

Test	Train		Experiment	F1-score (%)
	Real	Synthetic		
Coniferous-N	Real-D_2,2_	-	Training on all deciduous plots from scratch	18.1
	Real-D_2,2_	Sim_11,187_	Mixed training on 2 deciduous + all synthetic plots	67.2
Coniferous-C	Real-D_2,2_	-	Training on all deciduous plots from scratch	25.0
	Real-D_2,2_	Sim_11,187_	Mixed training on 2 deciduous + all synthetic plots	100.0
Coniferous-S	Real-D_2,2_	-	Training on all deciduous plots from scratch	7.1
	Real-D_2,2_	Sim_11,187_	Mixed training on 2 deciduous + all synthetic plots	56.7
Deciduous-R	Real-C_4,40_	-	Training on all coniferous plots from scratch	54.9
	Real-C_4,40_	Sim_11,187_	Mixed training on 3 coniferous + all synthetic plots	66.7
Deciduous-T	Real-C_4,40_	-	Training on all coniferous plots from scratch	36.8
	Real-C_4,40_	Sim_11,187_	Mixed training on 3 coniferous + all synthetic plots	41.7

### Procedural foliage generation parameters

Specifically, in each simulation: Initial Seed Density is the number of seeds placed along a 10-meter line. For example, a value of 3.0 means that $3^2 = 9$ seeds are placed per 10 m × 10 m area, approximately 900 seeds per hectare. Number of Steps is the number of simulation cycles used to spawn and thin the foliage. Average Spread Distance is the mean distance by which new seeds are dispersed from a parent tree in each cycle, and Spread Variance adds randomness to the spread distance for more natural variability.

In addition, another group of parameters controls the structure of the forest after spawning seeds: Collision Radius is the minimum distance required between the foliage instances to prevent overlap. This can be interpreted as the space needed for a tree to survive, depending on its size. Shade Radius is the area around each tree where its canopy suppresses the growth of new seedlings due to shading, and Max Age is the maximum number of simulation steps a foliage instance can endure before being removed. Furthermore, the foliage generation can be done for different groups of trees, e.g. overstory and understory, for a fine-grained modelling of the forest structures.

### Supplementary metrics for Section 4.1

Pretraining on our synthetic data allows the algorithm to achieve competitive performance after fine-tuning on only a few real forest plots, consistent across both coniferous (Table 10) and deciduous forest types (Table 11). With less than 0.1 ha of real data (the Real-C-S_1,1_), which represents only 2.2% of the 2.79 ha FOR-Instance dataset, the model pretrained on our synthetic data is still able to achieve competitive performance.

### Supplementary metrics for Section 4.2

Training with tree mesh point clouds (Nodal_4,20_) performs poorly, whereas UAV-LiDAR simulation (Sim_4,20_) significantly enhances performance (Table 12): F1 scores improve by over 30% for Coniferous-C, Coniferous-S, and Deciduous-R, and over 20% for other scenes.

In particular, the zero-shot performance of Sim_11,187_ exceeds training from scratch on few-shot datasets Real-C_3,3_ and Real-D_2,2_ for all scenes except Coniferous-S, where it is slightly worse (Table 10, Table 11).

### Mixed-training under domain shift setting

In Section 4.3, we observed that mixed training with the full real dataset (Real_6,42_) and synthetic data only matches reported SoTA performance without surpassing it. We hypothesise that this is because the test plots are drawn from the same regions as the training set, resulting in a small distribution shift that limits the benefit of synthetic data.

To test this hypothesis, we conduct mixed training experiments under a domain shift setting (Table 13), where the model is trained exclusively on one forest type (coniferous or deciduous) and evaluated on the other. In this cross-domain setting, adding synthetic data consistently mitigates the domain gap and significantly improves F1 scores. Notably, on Coniferous-C and Deciduous-R, mixed training achieves results comparable to or better than SoTA (F1: 100% and 66.7%, respectively). On other coniferous regions, mixed training with deciduous and synthetic plots yields a lower F1 than training on all real data. This is likely due to the heavy imbalance in the real dataset, which contains a disproportionately large number of coniferous plots (40 out of 42, with 37 from Coniferous-N alone).

These results confirm that the diversity and scale of synthetic data help bridge domain gaps. This also explains why synthetic pretraining is particularly effective in few-shot and zero-shot transfer settings, as demonstrated in the earlier experiments.

### Deciduous-T F1 Discrepancy in Section 4.3

In Table 8, we observed that our reproduced F1 score on the Deciduous-T test plot is substantially lower than the 69.4% reported by Xiang et al. (2024). Upon investigation, we find that this gap is not attributable to model quality but rather to the strong sensitivity of the F1 score to the IoU matching threshold in this specific case. In the following, we describe the inference pipeline, the underlying geometric cause, and a threshold ablation study.

**Inference pipeline and the IoU matching threshold.** ForAINet ($\mathcal {A}$) performs instance segmentation on large test plots via a cylinder-based sliding window. Inference proceeds mainly in three stages:

1. **Cylinder sampling.** The test plot is partitioned into overlapping cylinders (radius r=8 m) on a regular grid. Each cylinder is processed independently by $\mathcal {A}$ to produce per-instance clusters.
2. **Block merging.** Cylinder-level predictions are sequentially merged into a single global instance map. For each new cylinder, a predicted cluster is merged into an existing instance if their IoU exceeds 0.3; otherwise it is registered as a new instance.
3. **Plot-level evaluation.** A predicted instance is counted as a true positive (TP) if its IoU with any ground-truth (GT) instance exceeds the matching threshold τ (default: τ=0.5). The predictions below τ are false positives (FP); unmatched GT instances are false negatives (FN).

**Large tree crowns in Deciduous-T cause over-segmentation.** The Deciduous-T test plot contains 35 trees, including some with exceptionally large crowns. Table 14 compares the two deciduous plots used in our evaluation. Although the plots are of comparable size, Deciduous-T has substantially more crowns whose XY extent exceeds the radius of the cylinder (8 m). Consequently, these trees tend to span multiple adjacent cylinders, with their point clouds distributed across cylinder boundaries.

**Table 14:** Deciduous-T contains unusually large tree crowns that cause fragmented instance segmentation. Compared with Deciduous-R, Deciduous-T has fewer but larger deciduous crowns. Because instances are first segmented within individual cylinders (radius: 8 m) and then merged, trees crossing multiple cylinders are often split and may not be correctly merged into a single instance

	Deciduous-T	Deciduous-R
Plot extent (m)	33.7×49.2	28.5×25.6
Number of tree instances	35	64
Largest crown (m)	**15.5**	10.0
Trees with crown > 8 m	**7**	3
Trees with crown > 12 m	**4**	0

For such large crowns, the block-merging algorithm occasionally fails to consolidate all fragments belonging to the same GT tree. This occurs because the merging criterion is a local pairwise IoU threshold of 0.3: when a large crown spans several cylinders, the overlap between adjacent cylinder predictions of the same GT tree may not exceed this threshold, causing the tree to split into two or more fragmented instances. Since each fragment typically covers only ∼40–50% of the full GT tree, its IoU against the GT falls just below the matching threshold τ=0.5, causing it to be counted as a false negative rather than a true positive.

**Ablation on the IoU matching threshold** τ. To quantify this sensitivity, we recompute the Deciduous-T F1 score at varying values of τ, keeping the predicted and GT instance labels fixed. The results are shown in Table 15. Reducing τ from 0.50 to 0.35 cuts false negatives from 18 to 10, and the resulting F1 of 68.5% is within one percentage point of the 69.4% reported by Xiang et al. (2024). This confirms that borderline predictions with IoU just below 0.5 have a non-negligible effect on F1, since Deciduous-T contains only 35 GT trees and each such prediction shifts the score substantially. However, despite extensive investigations, we could not reproduce the exact number, likely due to undocumented implementation details or non-deterministic behavior in the original code that we could not fully control.

**Table 15:** Deciduous-T F1 is highly sensitive to the IoU matching threshold τ because of fragmented segmentations in this small plot (35 trees). Metrics are aggregated at the plot level across all cylinder samples. Predictions from the reproduction of Xiang et al. (2024) are fixed; only τ varies. Lowering τ from the default 0.50 to 0.40 reduces false negatives from 18 to 10, yielding an F1 of 68.5%, close to the 69.4% reported in Xiang et al. (2024)

τ	TP	FP	FN	Precision	Recall	F1-score
0.50	17	21	18	44.7	48.6	46.6
0.45	23	15	12	60.5	65.7	63.0
0.40	25	13	10	65.8	71.4	**68.5**
0.35	27	11	8	71.1	77.1	74.0
0.30	28	10	7	73.7	80.0	76.7

**Table 16:** A structurally diverse forest scene can be created using a small set of tree models. The Deciduous3 composition uses 2,053 tree meshes within a 250 m × 250 m plot, derived from 17 basic meshes with various canopy levels, as shown in this table

Mesh Name	Count	Percentage (%)
TreesAshLarge_A	60	2.92
TreesAshLarge_B	43	2.09
TreesAshLarge_C	42	2.05
TreesAshMedium_B	343	16.71
TreesAshMedium_C	180	8.77
TreesAshSapling_A	14	0.68
TreesAshSapling_B	32	1.56
TreesAshSapling_C	172	8.38
TreesLindenLarge_A	2	0.10
TreesLindenLarge_B	2	0.10
TreesLindenLarge_C	1	0.05
TreesLindenMedium_A	183	8.91
TreesLindenMedium_B	173	8.43
TreesLindenMedium_C	136	6.62
TreesLindenSapling_A	222	10.81
TreesLindenSapling_B	238	11.59
TreesLindenSapling_C	210	10.23

In Section 4.3, we aggregate predictions to the plot level to align with the evaluation protocol of Xiang et al. (2024) and enable direct comparison with their reported metrics. For reference, the mean F1 computed directly on all cylinder-level predictions is 61.4% regardless of the plot-level evaluation criterion, since the cylinder outputs are kept constant. This suggests that plot-level F1 can be sensitive to the choice of evaluation criterion in ways that do not reflect the underlying representation quality learnt from different datasets. In our few-shot experiments, we therefore report F1 as a direct mean over cylinder-level predictions without plot-level aggregation, which we believe more faithfully reflects the quality of the learnt representations.

### Supplementary table for Section 5.1

Table 16 shows the statistics of basic tree meshes used in the Deciduous3 forest scene content pack. The procedural foliage algorithm can create structurally realistic forest scenes at various canopy levels using these tree meshes. This method can enhance the generalisability of existing laser scans. For example, tree meshes can be derived from these scans to build a content pack or tree library, enabling the generation of forests with specific species compositions.

### Extended dataset with leaf–wood labels

Figure 10 shows the extended dataset with leaf and wood labels, the most fine-grained annotations achievable with current tree assets. These labels enable the leaf–wood separation task, which future researchers can use to train forest vision systems for quantitative studies of forest structural dynamics.
